# Genetic and structural validation of *Aspergillus fumigatus N*-acetylphosphoglucosamine mutase as an antifungal target

**DOI:** 10.1042/BSR20130053

**Published:** 2013-09-04

**Authors:** Wenxia Fang, Ting Du, Olawale G. Raimi, Ramón Hurtado-Guerrero, Karina Mariño, Adel F. M. Ibrahim, Osama Albarbarawi, Michael A. J. Ferguson, Cheng Jin, Daan M. F. Van Aalten

**Affiliations:** *Division of Molecular Microbiology, University of Dundee, DD1 5EH, Scotland, U.K.; †State Key Laboratory of Mycology, Institute of Microbiology, Chinese Academy of Sciences, Beijing 100101, People's Republic of China; ‡Division of Biological Chemistry and Drug Discovery, University of Dundee, DD1 5EH, Scotland, U.K.; §The College of Life Sciences Cloning Team, College of Life Sciences, University of Dundee, DD1 5EH, Scotland, U.K.

**Keywords:** cell wall, drug target, enzyme, inhibitor, nucleotide sugar, protein structure, *Af*AGM1, *A. fumigatus N*-acetylphosphoglucosamine mutase, AGM1, *N*-acetylphosphoglucosamine mutase, *Ca*AGM1, *Candida albicans* AGM1, Fru-6P, fructose 6-phosphate, G6PDH, glucose-6-phosphate dehydrogenase, GlcNAc, *N-*acetylglucosamine, GlcNAc-1P, *N-*acetylglucosamine-1-phosphate, GlcN-6P, glucosamine 6-phosphate, GFA1, glutamine: Fru-6P amidotransferase, GNA1, GlcN-6P acetyltransferase, IA, invasive aspergillosis, MIC, minimum inhibitory concentration, MM, minimal medium, RMSD, root mean square deviation, UAP1, UDP–GlcNAc pyrophosphorylase, UDP, uridine diphosphate

## Abstract

*Aspergillus fumigatus* is the causative agent of IA (invasive aspergillosis) in immunocompromised patients. It possesses a cell wall composed of chitin, glucan and galactomannan, polymeric carbohydrates synthesized by processive glycosyltransferases from intracellular sugar nucleotide donors. Here we demonstrate that *A. fumigatus* possesses an active *Af*AGM1 (*A. fumigatus N*-acetylphosphoglucosamine mutase), a key enzyme in the biosynthesis of UDP (uridine diphosphate)–GlcNAc (*N-*acetylglucosamine), the nucleotide sugar donor for chitin synthesis. A conditional *agm1* mutant revealed the gene to be essential. Reduced expression of *agm1* resulted in retarded cell growth and altered cell wall ultrastructure and composition. The crystal structure of *Af*AGM1 revealed an amino acid change in the active site compared with the human enzyme, which could be exploitable in the design of selective inhibitors. *Af*AGM1 inhibitors were discovered by high-throughput screening, inhibiting the enzyme with IC_50_s in the low μM range. Together, these data provide a platform for the future development of *Af*AGM1 inhibitors with antifungal activity.

## INTRODUCTION

*Aspergillus fumigatus* is a human fungal pathogen capable of causing infections ranging from allergic to invasive disease [1], and the major cause of IA (invasive aspergillosis) in immunocompromised patients [2]. In these patients, the crude mortality is 30–95% and remains about 50% even when treatment is given [3,4]. Antifungal drugs such as azoles, polyenes and candins are usually recommended for IA treatment [5]. However, new drugs are urgently needed due to the inefficacy, side effects and resistance that have emerged as important factors limiting successful clinical outcome [6,7].

Since the fungal cell wall is essential for viability and absent from the human cell, it has been recognized as an attractive target for the development of new antifungal agents. The core of the *A. fumigatus* cell wall is formed by a branched glucan–chitin complex, embedded in an amorphous ‘cement’ composed of linear chains of α-glucan, galactomannan and polygalactosamine [8]. Chitin, accounting for approximately 10–20% of the cell wall [9], is synthesized by chitin synthases that use UDP (uridine diphosphate)–GlcNAc as the sugar donor. In addition, UDP–GlcNAc is also utilized in the biosynthesis of cell wall mannoproteins and GPI (glycosylphosphatidylinositol)-anchored proteins [10,11].

In eukaryotes, UDP–GlcNAc (*N-*acetylglucosamine) is synthesized from Fru-6P (fructose 6-phosphate) by four successive reactions: (i) the conversion of Fru-6P into GlcN-6P (glucosamine 6-phosphate) by GFA1 (glutamine: Fru-6P amidotransferase); [12] the acetylation of GlcN-6P into GlcNAc-6P by GNA1 (GlcN-6P acetyltransferase); (*iii*) the interconversion of GlcNAc-6P into GlcNAc-1P (*N-*acetylglucosamine-1-phosphate) by AGM1 (*N*-acetylphosphoglucosamine mutase); and (*iv*) the uridylation of GlcNAc-1P into UDP–GlcNAc by UAP1 (UDP–GlcNAc pyrophosphorylase) [13]. The third enzyme, AGM1, is a member of the α-D-phosphohexomutase superfamily, that catalyses intramolecular phosphoryl transfer on a range of phosphosugar substrates [14]. AGM1 has been isolated and characterized from *Saccharomyces cerevisiae*, *Candida albicans* and *Homo sapiens* [15–18]. It has been reported that the AGM1 enzyme requires a divalent metal ion such as Mg^2+^ as a co-factor, but the reaction is inhibited by Zn^2+^ ions [19,20]. The sequence motif Ser/Thr–X–Ser–His–Asn–Pro is highly conserved and priming phosphorylation of the serine at the third position is required for full activity [15,21–23]. To date, only the crystal structure of *Ca*AGM1 (*Candida albicans* AGM1) has been reported, revealing four domains arranged in a ‘heart-shape’ [14]. The overall structure is similar to those of phosphohexomutases such as phosphoglucomutase/phosphomannomutase from *Pseudomonas aeruginosa* [24]. The *agm1* gene is essential for cell viability in *S. cerevisiae* [17]. Mice lacking the *agm1* homologue (*pgm3*) die prior to implantation, whereas heterozygotes have intrinsic haematopoietic and reproductive defects [25]. Although AGM1 has been proposed as a potential drug target, the issue of selectivity has not been explored and to date no drug-like inhibitor has been described for this class of enzyme.

Here, we show that *A. fumigatus* possesses a functional AGM1 enzyme that is essential for cell viability and cell wall synthesis. A crystal structure of the enzyme revealed the possible exploitable differences in the active site compared with the human enzyme. Using a high-throughput screening approach, we identified the first low micromolar inhibitors for this enzyme.

## MATERIALS AND METHODS

### Reagents, strains and growth conditions

Glc-1P (glucose-1-phosphate), Glc-6P (glucose-6-phosphate), G6PDH (glucose-6-phosphate dehydrogenase) from *Leuconostoc mesenteroides*, GlcNAc-6P, NAD^+^ and anthraquinone compounds were purchased from Sigma-Aldrich, UAP1 from *A. fumigatus* was a gift from Dr Ramón Hurtado-Guerrero, University of Dundee, UDP-Glc pyrophosphorylase from *Trypanosoma brucei* was a gift from Dr Karina Mariño, University of Dundee [26].

*A. fumigatus* strain KU80Δ*pyrG*^−^ derived from KU80Δ*pyrG*^+^ [27], a kind gift from Jean-Paul Latgé, Institut Pasteur, France, was propagated at 37°C on YGA (0.5% yeast extract, 2% glucose, 1.5% Bacto-agar) with addition of 5 mM uridine and uracil. The *Aspergillus nidulans alcA* promoter (P*_alcA_*) was induced by growing on the MM (minimal medium) [28] with 0.1 M glycerol, 0.1 M threonine or 0.1 M ethanol as carbon sources. YEPD (2% (w/v) yeast extract, 2% (w/v) glucose and 0.1% (w/v) peptone) medium and CM (complete medium) [29] were utilized to repress the P*_alcA_* completely and partially, respectively. Strains were grown in liquid medium at 37°C, with shaking at 200 rev./min. At the specified culture time point, mycelia were harvested, washed with distilled water, frozen in liquid N_2_ and then ground using a mortar and pestle. The powder was stored at −70°C for DNA, RNA and protein extraction.

Conidia were prepared by growing *A. fumigatus* strains on solid medium with or without uridine and uracil for 48 h at 37°C. The spores were collected, washed twice then resuspended in 0.1% (v/v) Tween 20 in saline solution, and the concentration of spores was confirmed by haemocytometer counting and viable counting.

### Cloning of *agm1*

The coding sequence of *Af*AGM1 (*A. fumigatus N*-acetylphosphoglucosamine mutase) (accession: XP_750370) was amplified by PCR from an *A. fumigatus* cDNA library (kindly provided by Jean-Paul Latgé, Institut Pasteur, France) using the forward primer P1 (5′- GC*GAATTC*ATGGCGTCTCCAGCCGTTCGC-3′) and the reverse primer P2 (5′- CT*GCGGCCGC*TTAAGAAGCCTGCAAGATTTCTTTGACGGTG-3′), exploiting the EcoRI and NotI restriction sites, for cloning into pGEX-6P1 (GE Healthcare) following a modification that removed the BamHI site from the original pGEX-6P1 vector such that the EcoRI site immediately followed the PreScission Protease coding sequence. The cloned protein sequence had a deletion of the amino acid residues VSSYGTFDGGMKGEFAD, corresponding to residues 85–101 of the reference XP_750370 sequence, but the protein sequence alignment with *Aspergillus clavatus* (XP_001269528) and *Neosartorya fischeri* (XP_001265046) *N*-acetylglucosamine-phosphate mutase sequences suggested that this deletion is most likely because of alternative splicing. All plasmids were verified by sequencing using the University of Dundee sequencing service.

### Construction of the conditional inactivation mutant

Plasmid pAL3 containing the P*_alcA_* and the *Neurospora crassa pyr-4* gene as a fungal selectable marker [30] was employed to construct a suitable vector allowing the replacement of the native promoter of the *A. fumigatus agm1* gene with the P*_alcA_*. To this end, an 898 bp fragment from −32 to +866 of *agm1* was amplified with primers P3 (5′-GG*GGTACC*ACACGACTTTCGCCAGGTC-3′, containing a KpnI site) and P4 (5′-GC*TCTAGA*TCCTTGCTCAGTAGGCTCAC-3′, containing an XbaI site). The PCR-amplified fragment was cloned into the expression vector pAL3 to yield pALAGM1N and confirmed by sequencing. The pALAGM1N was used to transform strain KU80Δ*pyrG*^−^ by PEG-mediated fusion of protoplasts [31] and positive transformants were selected by uridine/uracil autotrophy.

Genotyping of the transformants was performed by PCR and Southern blot analysis. For PCR analysis, three pairs of primers were employed. Primers P5 (5′- ATGGCGTCTCCAGCCGTT-3′) and P6 (5′- TTAAGAAGCCTGCAAGATTTC-3′) were used to amplify the *agm1* gene (2 kb). Primers P7 (5′-AAACGCAAATCACAACAGCCAAC-3′) and P8 (5′-CTATGCCAGACGCTCCCGG-3′) were used to amplify the *pyr-4* gene (1.2 kb). Primers P9 (5′- TCGGGATAGTTCCGACCTAGGA-3′) and P10 (5′- TGATGCCAATACCCATCCGAG-3′) were used to amplify the fragment from the P*_alcA_* to the downstream flanking region of the *agm1* gene (2.8 kb). For Southern blotting, genomic DNA was digested with PstI, separated by electrophoresis, and transferred to a nylon membrane (Zeta-probe^+^, Bio-Rad). The 898-bp fragment of *agm1* and a 1.2 kb HindIII fragment of the *N. crassa pyr-4* gene from pAL3 were used as probes. Labelling and visualization were performed using the DIG DNA labelling and detection kit (Roche Applied Science) according to the manufacturer's instructions.

### Quantitative PCR

Total RNA from the spores cultured in liquid MM was extracted using Trizol reagent (Invitrogen). cDNA synthesis was performed with 5 μg RNA using the SuperScript-First-Strand Synthesis System (Fermentas). Primers P11 (5′- TGTTGGAAGCTGAATGGGAAGC -3′) and P12 (5′-CGATCTCCTTAAC CAATTCGTCG -3′) were used to amplify a 96-bp fragment of *agm1*, and primers P13 (5′-CCACCTTGCAAAACATTGTT-3′) and P14 (5′-TACTCTGCATTTCGCGCATG-3′) were used for an 80-bp fragment of *tbp* gene (encoding TATA-box-binding protein). To exclude contamination of cDNA preparations with genomic DNA, primers were designed to amplify regions containing one intron in the gene [32,33]. Each PCR reaction mixture (20 μl) contained 8 μl sample cDNA, 0.4 μl ROX Reference Dye and 10 μl SYBR Premix Ex Taq™ from the SYBR Premix Ex Taq™ Kit (TAKARA), 0.8 μl ddH_2_O and 0.2 μM of each pair of primers. Thermal cycling conditions were 50°C for 2 min and 95°C for 1 min, followed by 40 cycles of 95°C for 5 s, 60°C for 60 s. Real-time PCR data were acquired using Sequence Detection software. The standard curve method [34] was used to analyse the real-time PCR data. Samples isolated from different strains and at different times were tested in triplicate.

### Electron microscopy and chemical analysis of the cell wall

To monitor the development of the cell wall structures, the conidia and mycelia grown in solid and liquid MM were fixed and examined with an H-600 electron microscope as described by Li et al. [35]. For the chemical analysis of the cell wall, conidia were inoculated into 100 ml MM or MMG liquid medium at a concentration of 10^6^ conidia ml^−1^ and incubated at 37°C with centrifugation (200 rev/min) for 36 h. The mycelium was harvested, washed with deionized water and frozen at −80°C. The cell wall components were isolated and assayed as described previously [36]. Three independent samples of lyophilized mycelial pad were used for cell wall analysis, and the experiment was repeated twice.

### A*f*AGM1 production and purification

LB medium (1 l) containing 0.1 mg ml^−1^ ampicillin was inoculated with 10 ml of an overnight culture of BL21 (DE3) pLysS cells harbouring the plasmid, and grown at 37°C to *A*_600_=0.8; at this absorbance, the temperature was reduced to 20°C and protein expression was induced by the addition of 250 μM IPTG (isopropyl β-D-thiogalactoside) and the incubation time was prolonged for a further 18 h. The cells were harvested by centrifugation at 3500 ***g*** at 4°C for 30 min, resuspended in Tris buffer (25 mM Tris, 150 mM NaCl, pH 7.5) containing lysozyme, DNAse (Sigma) and a tablet of protease inhibitor cocktail (Roche). Cells were lysed using a French press at 1000 psi. The insoluble fraction was removed by centrifugation at 40000 ***g*** for 30 min and the supernatant was incubated with Glutathione Sepharose 4B beads (GE Healthcare) previously equilibrated with the same buffer for 2 h. The beads were collected by centrifugation at 1000 ***g*** for 3 min and washed using the same buffer. The beads were then incubated with PreScission protease in the same buffer at 4°C on a rotating platform overnight. The cleaved protein was filtered from the beads, concentrated and confirmed by SDS–PAGE. In the last stage of purification, the protein was passed through a Superdex75 gel filtration column (2.6 × 60 cm) (Amersham Biosciences), previously equilibrated with 25 mM Tris buffer containing 150 mM NaCl, pH 7.5. Concentrated protein (5 ml) was loaded onto the column and eluted using the same buffer at 1.0 ml min^−1^ flow rate. Approximately 5 ml fractions were collected and fractions containing the protein were pooled and concentrated using a 10-kDa cut-off Vivaspin concentrator (GE Healthcare).

### Liquid chromatography-Tandem MS

Reduction and alkylation was performed on pure *Af*AGM1 protein prior to digestion by trypsin. The resulting peptides were dried down and reconstituted in 0.1% (v/v) formic acid. Peptides separated on a nano-C18 reverse phase column using a Dionex 3000 ultimate *n*-HPLC coupled to an LTQ-Orbitrap Velos mass spectrometer (Thermo Fisher). The mass spectrometer operated on a data-dependent CID mode, allowing automatic switching between MS, MS/MS and MS3 or MSA. The five most abundant ions from every full-scan over the range 340–1800 *m/z* at 60000 resolution in the Orbitrap analyzer were fragmented by MS/MS in parallel in the linear ion trap LTQ (MS/MS). Data were processed in Thermo Proteome Discoverer version 1.3 platform (Thermo Fisher Scientific Inc.), mass data were searched by MASCOT against ‘Uniprot_Swall _20120715’ protein database version 2.3, number of sequences 41660230, number of sequences after taxonomy: 207946; taxonomy specified: *Aspergillus*, with a Precursor Mass tolerance of 10 ppm and fragment mass tolerance of 0.6 kDa. Dioxidation (M), oxidation (M), phospho (STY) were allowed as variable modifications and carbamidomethyl (C) modification was fixed. The analysis layout included a phosphoRS node to calculate the probability of phosphorylation site mapping. The site mapping spectrum was manually inspected and validated.

### Protein crystallography

20 mg ml^−1^ of pure *Af*AGM1 protein in 25 mM Tris buffer, 150 mM NaCl with pH 7.5 was used to screen for crystals at 20°C using the sitting-drop vapour diffusion method. Each drop contained 0.6 μl of the protein solution with an equal volume of the mother liquor. To obtain the *Af*AGM1–Mg^2+^ complex, the protein was incubated at 4°C with 5 mM MgCl_2_ for 4 h before setting up crystal trays. The complex crystallized after 2–3 days in the space group P2_1_2_1_2_1_ (Table 3) from a mother liquor containing 0.1 M ammonium sulfate, 0.1 M sodium acetate trihydrate, pH 4.6. X-ray data from the *Af*AGM1 crystal were collected at the BM14 beam line of the European Synchrotron Radiation Facility (ESRF, Grenoble, France). Crystals were cryo-protected with 15% (v/v) glycerol in mother liquor and frozen in a nitrogen gas stream at 100 K. Data were processed with HKL2000 [37]. The structure of *Af*AGM1 was solved by molecular replacement using MOLREP [38] with the *Ca*AGM1 structure (PDBID 2DKA) [14] as the search model. Refinement was performed with REFMAC5 [39] and model building with COOT [40]. Pictures were generated using Pymol [41].

### Enzyme kinetics

Four methods were used to measure *Af*AGM1 activity. The first was a coupled assay with G6PDH as described by Liu et al. [42]. Briefly, the assay was carried out in a 100 μl reaction volume containing 50 mM MOPS pH 7.4, 1.5 mM MgSO_4_, 1 mM DTT (dithiothreitol), a range of concentrations of Glc-1P, 1 mM NAD^+^ and 0.01 units of G6PDH. The reaction was started by the addition of 10 nM *Af*AGM1 and incubated for 60 min at 20°C. The amount of NADH produced was measured using the micro plate fluorescence reader (FL×800).

A second assay involved UAP1 and pyrophosphatase as coupling enzymes, as described by Mok and Edwards [43]. The reaction mixture (100 μl) contained 50 mM MOPS pH 7.4, 1.5 mM MgSO_4_, 250 μM UTP, varying concentrations of GlcNAc-6P (2.5–300 μM), 100 nM *Af*AGM1, 0.5 μM *Af*UAP1 and 0.04 units pyrophosphatase to convert the *Af*UAP1 reaction product PPi to inorganic phosphate. The reaction was incubated at 20°C for 30 min and terminated by the addition of 100 μl Biomol green (0.03% (w/v) malachite green, 0.2% (w/v) ammonium molybdate and 0.5% (v/v) Triton X-100 in 0.7 N HCl) and left for a further 20 min at 20°C for colour development. Absorbance at 620 nm was read using a spectrophotometer.

A third assay involved coupling with UDP-glucose pyrophosphorylase using Glc-6P as the substrate, using the Biomol green assay as described [26].

A fourth assay was used to monitor product formation directly. The reaction mixture (100 μl) contained 50 mM MOPS buffer pH 7.4, 1.5 mM MgSO_4_, 1 mM Glc-1P and 20 nM *Af*AGM1. The reaction was incubated at 20°C for 30 min and terminated by adding 100 μl of 0.2 M NaOH. The samples were analysed by High Performance Anion Exchange Chromatography coupled to a Pulse Amperometric Detector (HPAEC-PAD, Dionex) using a CarboPac PA1 column and conditions adapted from Zhou et al. [44]. Briefly, a linear gradient from 150 mM sodium acetate (Merck), 0.1 M NaOH to 400 mM sodium acetate, 0.1 M NaOH was applied over 25 min, before lowering the concentration of sodium acetate back to the initial conditions over 5 min. The column was then re-equilibrated at 150 mM sodium acetate, 0.1 M NaOH for 5 min. The flow rate was kept constant at 0.25 ml min^−1^.

### Inhibitor screening

The Prestwick library (Prestwick chemical, France, 1120 compounds) and the LOPAC library (Sigma, 1280 compounds) were screened at 100 μM using the G6PDH coupled *Af*AGM1 assay. Compounds with percentage inhibition of ≥40% were investigated as possible hits. The compounds were purchased and false positives eliminated by testing inhibition of the coupling enzyme. IC_50_s of the most potent compounds against *Af*AGM1 were estimated using the direct assay method described above.

### *In vivo Af*AGM1 activity assay and MIC (minimum inhibitory concentration) assay

For *in vivo* protein extraction, the ground frozen powder was dissolved in 50 mM Tris–HCl pH 7.5 and placed on ice for 30 min. Intracellular proteins were collected by centrifugation. In order to eliminate PPi derived from the intracellular extract, a 10 kDa cut-off concentrator was used with each sample. Protein concentration was determined using the Folin–phenol method [45]. The *Af*AGM1 activity was determined as described by Mok and Edwards [43].

Three compounds were tested against *A. fumigatus* according to the Clinical and Laboratory Standards Institute (formerly NC-CLS) M38-A microdilution methodology. Briefly, conidial suspensions of 10^5^ ml^−1^ were dispensed (100 μl) into a microtiter plate containing serial two-fold dilutions of compounds. After incubating at 37°C for 48 h, growth was visually inspected. The MIC endpoint was defined as the lowest concentration producing complete inhibition of growth.

## RESULTS AND DISCUSSION

### *A. fumigatus* possesses a functional AGM1

**Figure 1:**
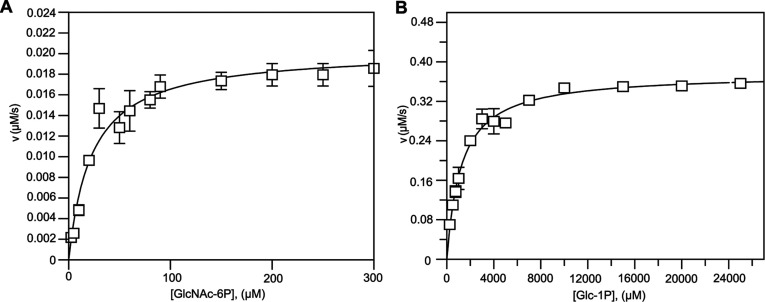
Steady-state kinetics of *Af*AGM1 Initial steady-state velocities are shown for *Af*AGM1 using different concentrations of (**A**) GlcNAc-6P, with *Af*UAP1 as coupling enzyme; (**B**) Glc-1P, with G6PDH as the coupling enzyme; details are described in the Materials and Methods section. The results are the mean±S.D. for three determinations.

A BLASTp search of the *A. fumigatus* genome revealed the existence of a putative *agm1* gene. The coding sequence of the gene was amplified by PCR from an *A. fumigatus* cDNA library, cloned into pGEX-6P-1 and overexpressed as a GST-fusion protein in *Escherichia coli*. Purification using glutathione beads followed by GST cleavage and size exclusion chromatography yielded 4 mg of pure *Af*AGM1 per litre of bacterial culture. A coupled assay with *A*. *fumigatus* UAP1 as the coupling enzyme was used to investigate the activity of *Af*AGM1 towards the predicted physiological substrate, GlcNAc-6P, yielding a *K*_m_ of 25±8 μM (Figure 1A, Table 1). This is comparable with the *K*_m_ of 46 μM obtained for *Hs*AGM1 for the same substrate [15].

**Table 1:** Kinetic parameters of *Af*AGM1 The coupled assay with G6PDH was used to measure *Af*AGM1 activity with Glc-1P as the substrate in the presence or absence of Glc-1,6-P_2_. The colorimetric assay involving purified *Af*UAP1 or *Trypanosoma brucei* UDP-glucose pyrophosphorylase as coupling enzymes was used to measure *Af*AGM1 activity with GlcNAc-6P or Glc-6P as the substrate. The results are the mean±S.D. for three determinations.

Substrate	*K*_m_ (μM)	*V*_max_ (μM s^−1^)	*k*_cat_ (s^−1^)	*k_cat_/K_m_* (μM^−1^ s^−1^)
Glc-1P	1200±100	0.38±0.01	37.6	0.0313
Glc-1P+Glc-1, 6-P_2_	400±30	0.41±0.01	41.0	0.1025
GlcNAc-6P	25±8	0.021±0.002	0.21	0.0084
Glc-6P	300±48	0.018±0.001	0.18	0.0006

Enzymes of the α-D-phosphohexomutase superfamily have been reported to be promiscuous in terms of their phosphohexose substrate specificity [22]. Indeed, *Af*AGM1 is active on Glc-1P as demonstrated with a different coupled assay, with G6PDH as a coupling enzyme, revealing a *K*_m_ of 1200±100 μM (Figure 1B, Table 1). This is different from the 12±1 μM *K*_m_ obtained for *Pa*PMM/PGM [46], suggesting *Af*AGM1 is more selective for GlcNAc phosphosugars. The presence of glucose-1,6-bisphosphate, an activator normally needed for this superfamily, did not enhance the activity implying that the enzyme may have been purified as the active, phosphorylated form [23]. *Af*AGM1 is also capable of converting Glc-6P into Glc-1P with a *K*_m_ of 300 μM, investigated using *T. brucei* UDP-glucose pyrophosphorylase as the coupling enzyme (Table 1). However, the *k_cat_*/*K_m_* values for GlcNAc-6P, Glc-1P and Glc-6P were 0.0084, 0.0313 and 0.0006 μM^−1^s^−1^, respectively, demonstrating that *Af*AGM1 has higher catalytic efficiency for Glc-1P than for GlcNAc-6P and Glc-6P (Table 1).

### *Af*AGM1 is essential for *A. fumigatus* survival

**Figure 2:**
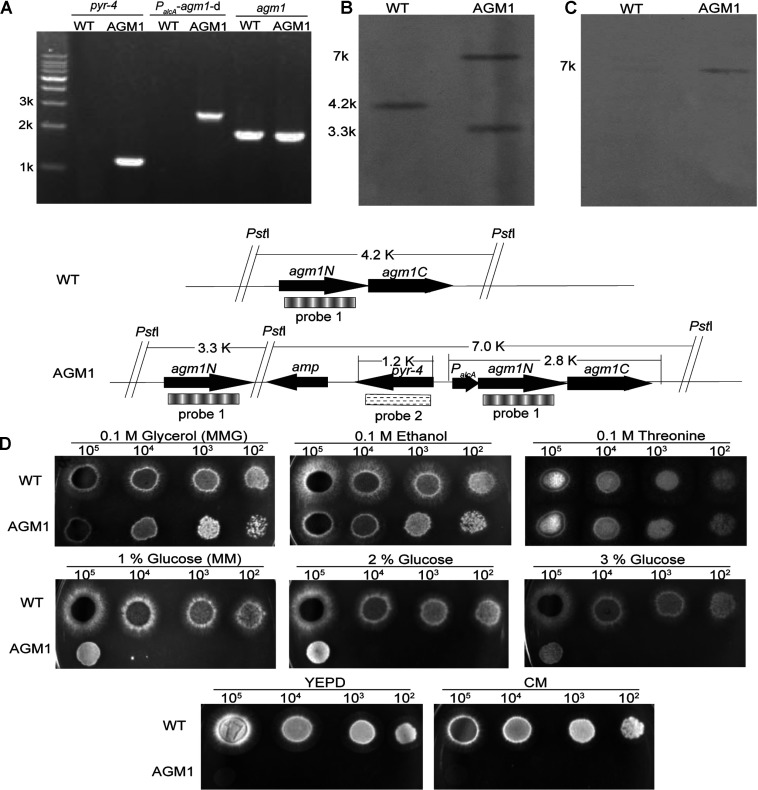
Generation of a conditional *agm1* mutant (**A**) PCR confirmation of the *agm1* mutant using primer pairs P5 and P6, P7 and P8, P9 and P10 to amplify the *agm*1 gene, the *pyr-4* gene and the fragment of *P_alcA_-* downstream of *agm1*, respectively. (**B**) Southern blot using an 898-bp fragment of the *agm1* gene as probe (probe 1). (**C**) Southern blot using a 1.2 kb HindIII internal fragment of the *N. crassa pyr-4* gene as probe (probe 2). (**D**) Growth of *A. fumigatus* strains on solid MM supplemented with 0.1 M glycerol, ethanol, threonine or 1, 2, 3% glucose, YEPD or CM, using serial dilutions from 10^5^–10^2^ conidia.

In fungi, AGM1 catalyzes an important step in the synthesis of UDP–GlcNAc, an important precursor for the synthesis of chitin, a key component of the fungal cell wall. Deletion of *AGM1* in *S. cerevisiae* has been shown to be lethal [13,17]. We first attempted to construct a deletion mutant in *A. fumigatus* by replacing the *agm1* gene with a *pyrG* gene. A total of 108 transformants were screened, none of them was positive. Therefore a conditional inactivation mutant was constructed by replacing the native promoter of the *agm1* gene with P*_alcA_*, a tightly regulated promoter that can be induced by ethanol, glycerol or threonine, and repressed completely on the YEPD medium [30,47]. To this end, a plasmid (pALAGM1N) that contains the *pyr-4* gene and P*_alcA_* fused to a 3′-end truncated version of the *agm1* gene was employed in transformation of *A. fumigatus* KU80Δ*pyrG*^−^ to generate a strain carrying the P*_alcA_*-*agm1* fusion gene by homologous recombination. One mutant named AGM1 was confirmed to be correct by PCR and Southern blot analysis. PCR analysis revealed that a 1217-bp fragment of *pyr-4* and a 2764-bp fragment of P*_alcA_*-*agm1* could be amplified from the mutant, while no such fragment was amplified from the wild-type strain (Figure 2A). When an 898-bp fragment of the *agm1* gene was used as probe, the expected 4.2 kb fragment was found in the wild-type, whereas the expected 3.3 and 7 kb fragments were detected in the AGM1 strain (Figure 2B). When the 1.2 kb fragment of the *pyr-4* gene was used as a probe, no fragment was found in the wild-type, whereas the expected 7 kb fragment was detected in the AGM1 strain (Figure 2C). These results demonstrated that the promoter of the *agm1* gene was replaced with the P*_alcA_* in the AGM1 strain.

The AGM1 strain grew normally on the solid MM containing 0.1 M glycerol (MMG), 0.1 M ethanol or 0.1 M threonine at 37°C for 36 h, whereas its growth was significantly inhibited on MM medium containing 1–3% glucose and completely retarded on YEPD or CM (Figure 2D), demonstrating that *Af*AGM1 is essential for *A. fumigatus* viability. Total suppression of *agm1* expression led to cell death. This suggests that no other members of the phosphohexomutase superfamily can substitute AGM1 in *A. fumigatus*, although the enzyme itself possesses both phosphoglucomutase and phosphoacetylglucosamine mutase activity (Figure 1 and Table 1).

In order to investigate the function of *Af*AGM1, MM containing 1% glucose (MM) was chosen for subsequent experiments. Under this condition, total RNAs were prepared from mycelia and the transcription levels of *agm1* in mutant and wild-type were examined by real-time quantitative PCR. Using relative standard curve quantitation, the transcription level of the *agm1* gene in the AGM1 strain was reduced to 32% of the wild-type transcript level. Intracellular proteins were extracted from mycelium cells and investigated for *Af*AGM1 activity using the *Af*UAP1 coupled assay revealing a 50% reduction in AGM1 activity.

### *Af*AGM1 is important for cell wall synthesis and ultrastructure

**Figure 3:**
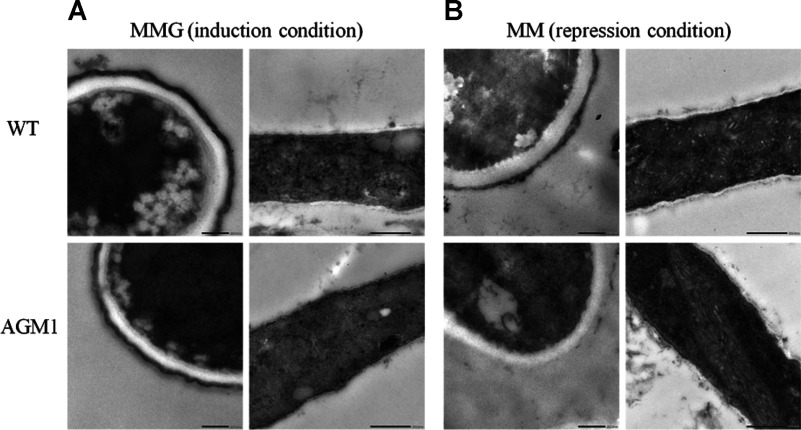
TEM spore and mycelia morphology for wild-type and *agm1* mutant strain Wild-type and strain AGM1 grown under (**A**) conditions of *agm1* induction and (**B**) conditions of *agm1* repression. Conidia and mycelia were collected after growing in solid or liquid MM and MMG media at 37°C for 36 h, fixed as described under the Materials and Methods section. The sections were examined with an H-600 electron microscope (Hitachi). Bar: 0.2 μm for conidia; 0.5 μm for mycelia.

Examination of the ultrastructure of the spore and hyphal cell wall revealed the spore of the AGM1 strain is similar to that of wild-type upon induction of *agm1* expression (using MM supplemented with 0.1 M glycerol, MMG) (Figure 3A). Using gene repression (with MM), the spore and hyphae of strain AGM1 had a thinner cell wall, unable to retain surface melanin (Figure 3B). Furthermore, the cell wall contents were analysed. With *agm1* induction, the cell wall components of the AGM1 strain were similar to those in wild-type. With *agm1* suppression, the content of α-glucan and β-glucan was increased by 25 and 33%, respectively; the amounts of glycoprotein and chitin in strain AGM1 were decreased by 16 and 19%, respectively; GlcNAc released from cell wall proteins was decreased by 34% and mannose was increased by 14% (Table 2). These results suggest that the suppressed expression of *agm1* induces a decreased content of chitin and GlcNAc in the *A. fumigatus* cell wall, presumably as a direct consequence of the decreased pools of the UDP–GlcNAc precursor. Although α/β-glucan contents were found to be increased (perhaps by the activation of the cell wall integrity signalling pathway [48,49]), this did not effectively compensate for the reduction of chitin, indicating that *Af*AGM1 is essential for cell wall synthesis in *A. fumigatus*.

**Table 2:** Chemical analysis of the cell wall Three aliquots of 10 mg lyophilized mycelia were used as independent samples for cell wall analysis, and the experiment was repeated twice. The values shown are microgram of cell wall component per 10 mg dry mycelia (±S.D.).

		Alkali soluble					Alkali insoluble	
		Mannoprotein					Chitin (μg)	β-glucan (μg)
Culture condition	Strain	Protein (μg)	GlcNAc (μg)	Gal (μg)	Man (μg)	α-glucan (μg)		
Induction (MMG)	WT	36±2	0.61**±**0.02	5.2**±**0.2	3.0**±**0.2	328**±**24	212**±**3	1137**±**57
	AGM1	39**±**2	0.64**±**0.04	5.3**±**0.3	2.9**±**0.3	322**±**22	230**±**6	1360**±**7
Repression (MM)	WT	51**±**3	0.61**±**0.02	8.1**±**0.3	4.9**±**0.2	587**±**45	194**±**2	1172**±**64
	AGM1	43**±**1	0.40**±**0.02	7.7**±**0.6	4.2**±**0.2	735**±**18	157**±**2	1554**±**4

### *Af*AGM1 possesses structurally exploitable differences compared with the human enzyme

**Figure 4:**
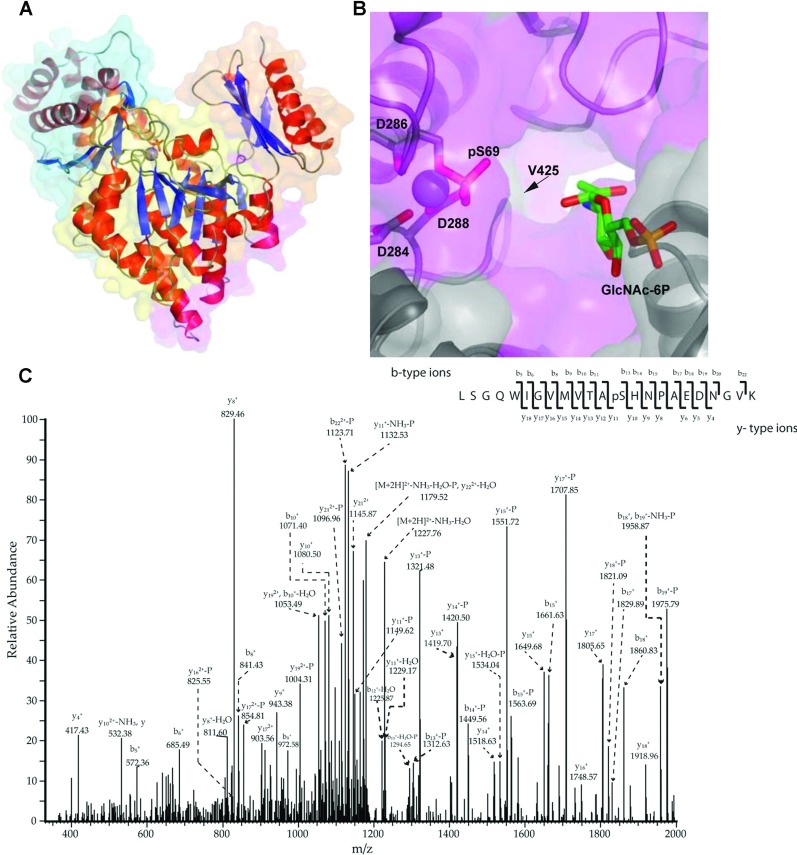
Crystal structure of *Af*AGM1 in complex with magnesium (**A**) A structural overview of *Af*AGM1. Domain I, II, III and IV are coloured in cyan, yellow, purple and orange, respectively. Secondary-structure elements of each domain are coloured red (helices) and blue (strands). The magnesium ion is shown as sphere. pSer69 is show as a sticks model. (**B**) Close-up view of the *Af*AGM1 active site, showing the magnesium ion (sphere), coordinating residues and pSer69 (sticks with grey carbon atoms). A model of GlcNAc-6P obtained by superposition with the *Ca*AGM1–GlcNAc-6P complex (PDBID: 2DKC [14]) is shown as sticks with green carbon atoms. A molecular surface is shown, coloured by sequence conservation with *Hs*AGM1 (purple: identical side chains, grey: non-identical side chains). (**C**) Annotated MS/MS spectrum of an *Af*AGM1 tryptic peptide covering the Ser69 phosphorylation site. On the top of the panel an extensive coverage b- and y-type ions of the peptide fragment ions shown made the site of phosphorylation localization to be precisely determined on Ser^69^. This manual assignment was reinforced by the bioinformatics tool ‘phosphoRS’ a phosphorylation-site localization probabilities software calculator ‘phosphoRS’ version 1.3.0.339 (Thermo Fisher Scientific Inc.) results shown on top over the peptide sequence.

The *agm1* gene disruption provides genetic validation of *Af*AGM1 as a potential antifungal target in *A. fumigatus*, justifying efforts towards the discovery of inhibitors of this enzyme. Although the structure of human AGM1 has not been reported, this enzyme is 49.3% identical to *Af*AGM1 at the amino acid sequence level. Given that mice lacking the *agm1* orthologue die prior to implantation [25] it is essential to discover inhibitors that selectively inhibit the fungal enzyme over the human orthologue. To investigate possible differences in the active site compared with the human enzyme, the crystal structure of *Af*AGM1 in complex with magnesium was determined to 2.35 Å resolution (Table 3), with two protein molecules in the asymmetric unit. The molecules interact via a 553 Å^2^ contact surface area, suggesting weak crystallographic (rather than physiologically relevant) contacts, in agreement with the gel filtration trace that showed a monomer in solution. The overall structure of this enzyme is similar to that of *Ca*AGM1 [14] (51.6% sequence identity, RMSD (root mean square deviation) of 1.2 Å on 496 Cα atoms) and the *Pa*PMM/PGM [24] (19.6% sequence identity and RMSD of 2.3 Å on 343 Cα atoms). Like other members of this superfamily, *Af*AGM1 consists of four domains (Figure 4A) forming a heart shape. Domain 1 (residues 1–187) bears the predicted active serine loop, domain 2 (residues 188–305) bears the metal-binding loop, domain 3 (residues 306–442) bears the sugar-binding loop and domain 4 (residues 443–542) bears the phosphate-binding loop. Although the *Af*AGM1 structure was determined in absence of any substrates or products, all amino acids important for substrate binding and catalysis, as gleaned from the *Ca*AGM1 structure, are conserved, in agreement with the observed catalytic activity of *Af*AGM1 (Figure 4B). Strikingly, the electron density revealed a phosphorylated active site S69 (Figure 4A), which was confirmed separately by mass-spectrometric phosphosite mapping (Figure 4C), explaining why the enzyme is active in absence of glucose-1,6-bisphosphate (Table 1), an activator normally required to load the active site of this class of enzyme with a phosphate [23,46,50]. It is probably that *Af*AGM1 became phosphorylated during expression in the *E. coli* host. It is known that enzymes of the wider family of phosphohexomutases are magnesium-dependent. In *Af*AGM1, the magnesium ion is pentagonally coordinated in a square-pyramidal arrangement by pSer69, Asp^284^, Asp^286^ and Asp^288^ (Figure 4B). This type of coordination has also been described for the structure of *Ca*AGM1 complexed with either GlcNAc-1P or GlcNAc-6P and Zn acting as an inhibitor [14].

**Table 3:** Details of diffraction data collection and structure refinement Values between brackets are for the highest resolution shell. All measured data were included in structure refinement.

Measurement	*Af*AGM1+Mg^2+^
Resolution	25.00 (2.35)
Space group	*P*2_1_2_1_2_1_
Unit cell	
* a* (Å)	74.7
* b* (Å)	86.6
* c* (Å)	185.4
No. of reflections	183592
No. of unique reflections	48393
*I*/σ (*I*)	34.7 (1.8)
Completeness (%)	99.87
Redundancy	3.5 (3.9)
*R*_merge_ (%)	5.7 (66.6)
RMSD from ideal geometry	
Bond distance (Å)	0.010
Bond angle (°)	1.39
*R*_work_ (%)	21.8
*R*_free_ (%)	27.7
No. of residues	987
No. of water mol.	38
B factors (Å^2^)	
Overall	18.9
Protein	17.8
Ligand	53.3

Although the catalytic machinery is fully conserved, careful analysis of sequence conservation in the active site area revealed that the human and fungal enzymes possess differences near the substrate-binding site (Figure 4B). For example, within the loop carrying the catalytic serine, *Af*AGM1 Ala^73^ is equivalent to Glu^68^ in the human enzyme. The sugar phosphate-binding loop harbours Ala^506^ and Ala^512^ in *Af*AGM1, equivalent to Pro^497^ and Val^503^ in the human enzyme, respectively. Within the active site itself, located close to the phosphoGlcNAc binding site, Val^425^ occupies a position in *Af*AGM1 that is equivalent to the smaller Ala^416^ in the human enzyme (Figure 4B). Such differences can be exploited in the design of inhibitors to selectively target the fungal enzyme.

### Screening-based discovery of micromolar *Af*AGM1 inhibitors

**Figure 5:**
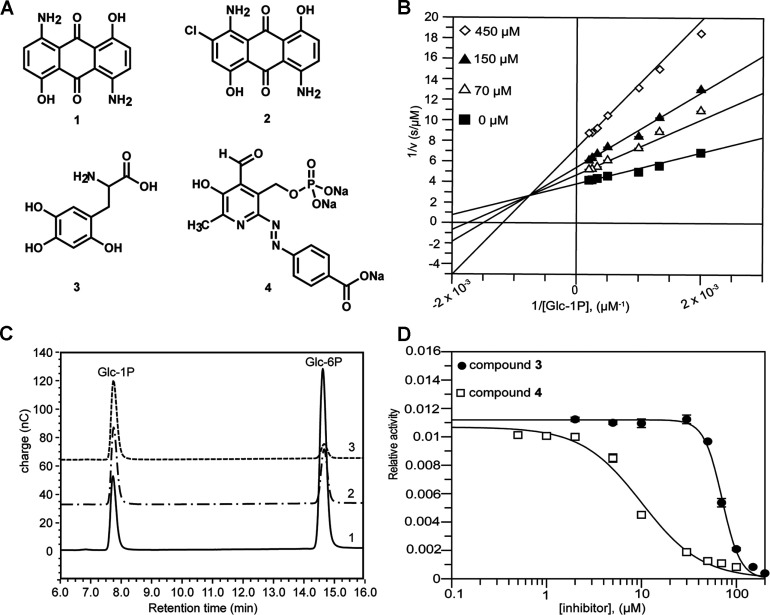
Inhibitors of *Af*AGM1 (**A**) Structures of the *Af*AGM1 inhibitors identified by high-throughput screening. **1**: 1,5-diamino-4,8-dihydroxyanthraquinone, **2**: 2-chloro-1,5-diamino-4,8-dihydroxyanthraquinone (Disperse Blue 56), **3**: 6-hydroxy-DL-DOPA, **4**: (tri-sodium 4-[(2z)-2-[4-formyl-6-metyl-5-oxo-3-(phosphonatooxymetyl)-pyridin-2-ylidene] hydrazinyl] benzoate). (**B**) Lineweaver–Burk plot of inhibition of *Af*AGM1 by compound **1**, giving a *K*_i_ of 300±13 μM. (**C**) HPAEC-PAD chromatogram for the direct assay of Glc-1P and Glc-6P showing inhibition of *Af*AGM1 activity; 1. The positive control (without inhibitor); 2. Reaction containing 100 μM compound **3**; and 3. Reaction containing 100 μM compound **4**. (**D**) IC_50_ curves of compounds **3** and **4** inhibition of *Af*AGM1 using the HPAEC-PAD assay and 1 mM Glc-1P substrate.

To identify potential inhibitors of *Af*AGM1, high-throughput screening of the Prestwick (1120 compounds) and Sigma LOPAC (1280 compounds) libraries was carried out at 100 μM compound concentration. Screening was performed using the G6PDH coupled assay. Compounds with percentage inhibition of ≥ 40% were considered to be hits, corresponding to 84 compounds (3.5%) of the total screened. These compounds were tested against the coupling enzyme, resulting in a pool of 16 true positive hits. Where possible, structural representatives of different scaffolds were purchased and retested on *Af*AGM1. A group of anthraquinone-based compounds were found to be the most potent *Af*AGM1 inhibitors identified from the Prestwick screen with 1,5-diamino-4,8-dihydroxyanthraquinone (Figure 5A, compound **1**) having a *K_i_* of 300±13 μM by a non-competitive mixed inhibition mechanism (Figure 5B). Interestingly, a similar compound, the organic dye Disperse Blue 56 (2-chloro-1,5-diamino-4,8-dihydroxyanthraquinone) (Figure 5A, compound **2**) was identified by virtual screening method as an inhibitor of *Pa*PMM/PGM with an IC_50_ of 5 μM [42]. The authors observed that inhibition of this enzyme was due to aggregation of the compound. However, no inhibition of *Pa*PMM/PGM was observed when the compound **1** was tested [42]. The selectivity of this compound may be the result of the dissimilarity between *Af*AGM1 and *Pa*PMM/PGM (sequence identity of only 19.6%).

The other compounds, compound **3** (6-hydroxyl-DL-DOPA) and compound **4** (tri-sodium 4-[(2z)-2-[4-formyl-6-metyl-5-oxo-3-(phosphonatooxymetyl)-pyridin-2-ylidene] hydrazinyl] benzoate) (Figure 5A) from the LOPAC screen were tested against *Af*AGM1 by a direct assay method using HPAEC-PAD (Figure 5C) to avoid the observed slight inhibition of the coupling enzyme and were found to inhibit *Af*AGM1 activity with IC_50_s of 58±4 μM and 7.1±0.2 μM for the compounds **3** and **4**, respectively (Figure 5D). When compounds **1**, **3** and **4** were tested on *A. fumigatus* cultures, compounds **1** and **3** showed severe precipitation during dilution for the MIC assay, whereas compound **4** did not inhibit growth of either the wild-type or AGM1 mutant strains at concentrations up to 1.4 mM. Although these compounds are not active against *A. fumigatus*, either because of the solubility of the compounds, limited cell penetration or low efficacy, they are the first low micromolar inhibitors identified for the phosphohexomutase superfamily. Structural complexes of these inhibitors with *Af*AGM1 and synthesis of derivatives to address solubility, penetration and potency may lead to insights into mode of action and generation of molecules reproducing the genetic phenotype of the *agm1* gene knockout.

In conclusion, by combination of genetic and structural approaches we have validated *Af*AGM1 as a potential antifungal drug target. Together with the novel compounds identified here, these results provide a platform for the development of AGM1 inhibitors that target fungal cell wall synthesis.
